# Notes and News

**Published:** 1896-06-13

**Authors:** 


### Notes and News.

(Collected and Communicated.)

An Exhibition of Manufactures, Appliances, and Inventions for the Saving of Life is to be held at the Central Hall, Holborn, from July 9th to 18th next, in aid of the Guy's Hospital Fund. Demonstrations by members of the St. John's Ambulance and the Life Saving Society will be given, and many other attractions will be offered.

The death of Sir George Johnson, M.D., F.R.S., at his residence in Savile Row, on June 3rd, has removed from our midst a well-known figure. For many year he was physician to King's College Hospital, and will long be remembered for his investigations regarding the nature of diseases of the kidney and his views as to the treatment of cholera.

We are glad to learn that Miss Sharman has succeeded in securing a seaside home for the orphans she has under her charge. The house, which is at Hastings, is bought and paid for, and kind friends are coming forward to help with the furnishing. Miss Sharman's scheme is an admirable one, and her careful and energetic administration deserves every encouragement.

We hope next week to give a full account of the proceedings at the dinner and reception at the Imperial Institute, on behalf of Guy's Hospital, on the 10th instant. H.R.R. the Prince of Wales has taken a most genuine and active interest in the matter; he has been well seconded in his efforts by several of the governors, notably by Mr. Cosmo Bonsor, M.P., and by Dr. Perry and Mr. Fripp, F.R.C.S., the honorary secretaries, whose services have been invaluable. We are confident that the amount raised at this dinner, which takes place after we go to press, will constitute a record and be memorable in the annals of charity throughout the world.

A lady, who wished her gift to be anonymous, has bequeathed £1,300 to the Dover Infectious Diseases Hospital, which was built and presented to the town by a local physician, Dr. Astley.

The medical profession appears to be particularly popular in France, judging from the fact that medical men hold the office of mayor in different Departments of the country.

Purses of five guineas and upwards are to be presented to H.R.H. the Duchess of York on the occasion of the fête to be held on July 1st at the Middlesex Hospital, in aid of the convalescent home in connection with the hospital.

An out-patient department has been opened at the Newcastle Hospital for Sick Children. It has been built by Lord Armstrong in memory of the late Lady Armstrong, and presented to the Fleming Memorial Hospital. The building is of red Cheshire bricks with stone dressings, in seventeenth century style.

A meeting in favour of the Metropolitan Hospital Saturday Fund was held last week at Hackney, Lord Russell of Killowen presiding. The meeting was held with the principal object of increasing the interest of the working classes in the Fund, the project meeting with hearty support.

The Birmingham Hospital Saturday collection has already exceeded £12,000, and therefore this year's collection may fairly be expected to exceed all previous ones. Contributors have just received further encouragement in supporting the useful scheme by the gift to the Fund of a convalescent home for children, thus making the accommodation for patients recovering from illness complete. The new home, situated half-way between the existing convalescent institutions belonging to the Fund, has been presented by an anonymous donor. The house is beautifully situated, and is now having the necessary alterations and additions made, and will, it is hoped, be ready for occupation the middle of July.

We hope the Hospital Sunday Supplement which we publish with to-day's issue may prove as fruitful in results for the benefit of the voluntary hospitals as that of last year. It is by no means easy to bring original treatment to bear upon the same subject year after year, and the production of this Supplement entails an appreciable amount of devotion and enthusiasm on the part of members of the staff which all must value for its own sake. This year the hospitals are specially indebted to a hard-worked and able artist, Mr. H. M. Paget, who has spontaneously declined to accept any fee for the excellent illustrative work which he has done on behalf of all the hospitals. Mr. Paget desired to make this gift of personal service to the cause of the hospitals—a gift of very real value—at a most busy time of the year, which entailed an "all-night sitting" upon him to do the drawings, owing to great pressure of other work. Would that every Londoner would go and do likewise in some equivalent manner on this Hospital Sunday!

